# RadShield: semiautomated shielding design using a floor plan driven graphical user interface

**DOI:** 10.1120/jacmp.v17i5.6287

**Published:** 2016-09-08

**Authors:** Matthew C. DeLorenzo, Dee H. Wu, Kai Yang, Isaac B. Rutel

**Affiliations:** ^1^ Department of Radiological Sciences University of Oklahoma Health Science Center Oklahoma City Ok; ^2^ Division of Diagnostic Imaging Physics, Department of Radiology Massachusetts General Hospital Boston MA USA

**Keywords:** RadShield, shielding design, radiation protection, radiation shielding, GUI

## Abstract

The purpose of this study was to introduce and describe the development of RadShield, a Java‐based graphical user interface (GUI), which provides a base design that uniquely performs thorough, spatially distributed calculations at many points and reports the maximum air‐kerma rate and barrier thickness for each barrier pursuant to NCRP Report 147 methodology. Semiautomated shielding design calculations are validated by two approaches: a geometry‐based approach and a manual approach. A series of geometry‐based equations were derived giving the maximum air‐kerma rate magnitude and location through a first derivative root finding approach. The second approach consisted of comparing RadShield results with those found by manual shielding design by an American Board of Radiology (ABR)‐certified medical physicist for two clinical room situations: two adjacent catheterization labs, and a radiographic and fluoroscopic (R&F) exam room. RadShield's efficacy in finding the maximum air‐kerma rate was compared against the geometry‐based approach and the overall shielding recommendations by RadShield were compared against the medical physicist's shielding results. Percentage errors between the geometry‐based approach and RadShield's approach in finding the magnitude and location of the maximum air‐kerma rate was within 0.00124% and 14 mm. RadShield's barrier thickness calculations were found to be within 0.156 mm lead (Pb) and 0.150 mm lead (Pb) for the adjacent catheterization labs and R&F room examples, respectively. However, within the R&F room example, differences in locating the most sensitive calculation point on the floor plan for one of the barriers was not considered in the medical physicist's calculation and was revealed by the RadShield calculations. RadShield is shown to accurately find the maximum values of air‐kerma rate and barrier thickness using NCRP Report 147 methodology. Visual inspection alone of the 2D X‐ray exam distribution by a medical physicist may not be sufficient to accurately select the point of maximum air‐kerma rate or barrier thickness.

PACS number(s): 87.55.N, 87.52.‐g, 87.59.Bh, 87.57.‐s

## I. INTRODUCTION

Diagnostic medical X‐ray exams produce ionizing radiation which must be shielded to protect radiation workers and the general population. The National Council on Radiation Protection (NCRP) has provided guidelines to the medical physics community for shielding radiographic and fluoroscopic rooms (R&F rooms) contained in NCRP Report 147.^(1)^ NCRP Report 147 has since been the professionally recognized reference for shielding radiographic and fluoroscopic rooms.^(2)^ Such guidelines are followed by medical physicists to recommend minimum necessary barrier thicknesses to attenuate primary and scattered radiation to meet their room specific design goals. Based on the calculated results, the medical physicist typically rounds up to the nearest prefabricated slabs of lead thickness in 0.079 cm increments.

In this work, a graphical user interface was developed to accurately perform radiation shielding design pursuant to NCRP Report 147. The software, RadShield, is a Java‐based drawing program designed to both accurately compute barrier thickness and give aesthetic presentation of results. This software package now provides an opportunity to perform shielding calculations for more than one calculation point at a time. RadShield produces more thorough and rigorous shielding designs than a physicist doing calculations by hand or spreadsheet because it computes required barrier thicknesses using an iterative method for hundreds or thousands of points and reports the maximum values for each barrier.

Example calculations in NCRP Report 147 encourage overconservative shielding for the sake of increased safety and simplicity.^3^, ^4^, ^5^ Due to the fact that it is impractical to perform an exhaustive evaluation of barrier thickness at more than a handful of points, they tend to err on the side of overconservative shielding.^3^, ^6^ This would ensure safety to the general public and hospital staff, but it could cost the radiology department or hospital more money than is necessary to meet regulatory standards.^(5)^

The workload distribution and transmission curves for diagnostic range primary and secondary radiation, found by Archer et al.^(7)^ and consolidated by Simpkin^(8)^ based on clinical energy spectra for common exam distributions, are included in the formulation of NCRP Report 147. The energy spectra of the exam types can vary greatly. For instance, primary transmission for a radiographic chest exam is far greater than the secondary transmission for a fluoroscopic tube. Both chest exams and fluoroscopic exams commonly take place in the same room and will result in a mixture of X‐ray spectra incident on all barriers averaged over one week or one year. NCRP 147 suggests that the physicist use the transmission curve for the most penetrating X‐ray spectrum to calculate barrier thickness. This is a conservative assumption meant to simplify the hand calculation of the medical physicist, but simultaneously introduces error. A more complete shielding design would take all of the workload distributions present in the exam room into consideration rather than assuming the entire unshielded air‐kerma rate incident on the barriers comes from the most penetrating workload distribution.

A review of the literature suggests that only XRAYBARR, a shareware program by Doug Simpkin,^(9)^ solves for air‐kerma rate and barrier thickness iteratively for diagnostic radiation shielding. XRAYBARR accounts for multiple workload distributions, outputs organized results, and is an improvement over hand calculations and spreadsheets. However, Simpkin's program calculates air‐kerma rate and barrier thickness at only one point for which the user must manually locate, then measure and enter distances. XRAYBARR also doesn't take into account the entire floor layout and line of sight geometry when performing calculations. It is still incumbent on the physicist to know which point on the floor plan requires the greatest amount of barrier thickness. The focus of this work is to make shielding design more accurate and less prone to manual error by using an iterative approach and incorporating geometrical information from the floor plan, so many points are tested and the true maximum value of shielding thickness is used.

**Figure 1:**
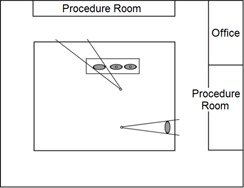
Modern radiographic and fluoroscopic (R&F) room with example spatial arrangement of exam types. Each exam has its own number of patients and workload distribution. Circles indicate X‐ray source locations and ellipses indicate patient location.

In shielding design, one must account for a variety of diagnostic exams taking place in a typical radiographic and fluoroscopic room (R&F room) (Fig. 1). For example, chest exams and some spine exams are performed in the R&F room with a standing patient and the X‐ray source facing the wall. Other exams are performed with the patient prone or supine on a radiographic table. Cross table exams may make use of oblique angles. Fluoroscopic exams use a continuous or pulsed X‐ray beam to view patient anatomy in real time. Given that modern R&F rooms have a variety of patient and X‐ray source locations, it is the responsibility of the medical physicist to take the contributions of each exam type used in the clinic into consideration when calculating air‐kerma rates and ultimately barrier thicknesses.^1^, ^7^, ^8^ As more exam types are considered in the shielding design, the complexity of the project increases^(10)^ and it becomes more time‐consuming for the physicist to measure distances and calculate by hand or spreadsheet.^(11)^ In some cases, the physicist might calculate barrier thickness based on a room that isn't adjacent to the R&F room with a lower unshielded air‐kerma rate.^(1)^ For example, uncontrolled offices away from the R&F room with design goals of 0.02 mSv per week^(1)^ may contain the most stringent point for calculating barrier thickness. RadShield introduces the capacity to perform multiple point calculations within nonadjacent rooms of interest, while the current standard of shielding design relies on the physicist to understand the floor plan layout well enough to accurately find the most stringent point *a priori*. The medical physicist will round to the next thickest commercially available lead sheet (generally 0.079 cm). Differences of a few percent may not matter in some cases, but calculating barrier thickness as precisely as possible is the ideal starting point for recommending final thickness.

## II. MATERIALS AND METHODS

### A. RadShield approach

**Figure 2:**
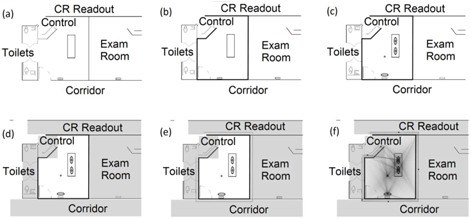
Overview of RadShield approach: (a) the user first imports an image file to the workspace and defines the scale in terms of pixels per meter; (b) the scaled floor plan is used to draw barriers in their correct locations; (c) X‐ray tube locations are drawn for different exam types; (d) occupied regions are drawn for which design goals and occupancy factors are specified; (e) once walls, tubes, and regions are drawn, sample points are created 0.3 m from the barriers indicating where calculations will take place; (f) the contributions from all X‐ray sources and patient locations are summed to find the air‐kerma rate and barrier thickness at every sample point. The maximum values for each barrier are displayed in a separate window.

RadShield is a Java‐based GUI that is designed to be used through a series of ordered steps (Fig. 2). The GUI permits the user to enter an image of the X‐ray room floor plan from disk in .png, .jpg, .tif or .gif format to be overlaid on the workspace. This enables the user to adapt the floor plan to match the correct dimensions of the exam room. The user then sets the scale of the image, in terms of pixels per meter. This scaled image file serves as a template over which the physicist draws barriers, regions of interest, and X‐ray sources in their appropriate locations. Shielding variables, such as number of exams, workload distribution, and source‐to‐patient distance, are entered for each drawn object through a GUI‐driven menu‐based interface. NCRP 147 suggests that the physicist perform calculations 0.3 m beyond the barrier. For each user‐drawn barrier, a parallel line is created automatically 0.3 meters beyond it, on the opposite side of an X‐ray source location, and is sampled along its length (Fig. 2(e)). If there are X‐ray sources on both sides of a barrier, two parallel lines are created and sampled on either side. The distance between the separation line and the barrier, as well as the density at which it is sampled, may be increased or decreased by the user.

When all required information has been entered, the user presses a final button to run the calculations. Air‐kerma rate and barrier thickness are calculated at multiple points beyond barriers and within nonadjacent regions of interest. Once the results are generated, the design and shielding variables may be saved in .xml format to disk and loaded at a later time for modification.

For each sample point, a black circle appears showing that it is a point for which barrier thickness was calculated. The points at which the maximum value for air‐kerma rate and barrier thickness are found are displayed on the workspace as large red and blue circles respectively. The maximum found numerical values for air‐kerma rate and barrier thickness are displayed on a separate window below the canvas which updates if the user makes a change and recalculates.

The air‐kerma rate contributions from all X‐ray tubes and patient locations are summed at each sample point. The total air‐kerma rate at each sample point is stored in a wall‐specific array and the maximum value for each wall is reported to the user once the calculation is completed. NCRP 147 contains example calculations which use the total air‐kerma rate and the most penetrating workload distribution to obtain final barrier thickness. RadShield finds the barrier thickness required to attenuate the user specified workload distributions to the occupancy adjusted design goal (P/T).^1^, ^10^, ^12^

RadShield calculates barrier thickness using an iterative method which employs the barrier transmission factor for each X‐ray source, as described by Simpkin.^1^, ^7^ NCRP Report 147 gives logarithmic fitting parameters, α, β, and γ for lead, concrete, gypsum wallboard, wood, steel and glass for different workload spectra.^2^, ^8^, ^13^ Fitting parameters for 150 kVp primary radiation are used for leakage radiation as described in Appendix C of NCRP Report 147. The first step in the method is to define upper and lower limits for calculated material thickness. Zero (mm) of material is used as the lower limit and a very large, arbitrary thickness, x, as the upper limit. The second step is to calculate the transmission factor (B) for each air‐kerma rate contribution incident on the upper limit of the barrier thickness, x. To find the transmission factor as a function of thickness, the transmission factor Eq. 4.5 from NCRP 147 is used:

(1) $$B(x)=\frac{Pd^{2}}{K^{1}NT}$$

where *P* is the design goal (${\text{mGy cm}}^{-1}$), *T* is the room‐specific occupancy factor, $K^{1}$ is the air‐kerma rate at one meter (mGy), *N* is the number of exams per week (${\text{wk}}^{-1}$) for a particular exam, and *d* is the distance (m) from the X‐ray source or scattering body to the calculation point.

When taking radiation line of sight path length through the material into account (for an angle of incidence θ, with respect to the normal), NCRP Report 147 Eq. 4.6 becomes:

(2) $$x=\frac{1}{\alpha \gamma }ln\left(\frac{B^{-\gamma }+\frac{\beta }{\alpha }}{1+\frac{\beta }{\alpha }}\right)\text{cos}\;\theta$$

The transmission factor of the ith X‐ray exam as a function of barrier thickness can be found by rearranging the above equation.^(14)^

(3) $$B_{i}={\left[\left(1+\frac{\beta_{i}}{\alpha_{i}}\right)e^{\alpha_{i}\gamma_{i}x/\text{cos}\;\theta_{i}}-\frac{\beta_{i}}{\alpha_{i}}\right]}^{-\frac{1}{\gamma_{i}}}$$

Care must be taken when using this path length correction for scatter generating materials such as concrete.^(15)^ Each workload distribution has a corresponding transmission factor for a given thickness of material. The third step is to multiply the incident air‐kerma rate contribution for each exam type by its corresponding transmission factor.

(4) $$K_{\text{trans}}=\sum_{i=1}^{N_{\text{exams}}}B_{i}K_{i}$$

The transmitted air‐kerma rates are summed and compared to (P/T). Ideally, the transmitted air kerma rate is equal to P/T so that the exact amount of material is calculated to meet the design goal. RadShield adjusts the barrier thickness using a bisection method,^(16)^ and repeats the transmission factor and transmitted kerma calculations until the barrier thickness converges to a value satisfying the following conditions:

(5) $$K_{\mathrm{trans}}–\frac{P}{T}\leq 0\;\&\;\left|K_{\mathrm{trans}}-\frac{P}{T}\right|<10^{-14}$$

**Figure 3:**
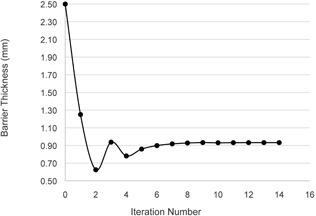
Convergence of barrier thickness by iteration. The first iteration assumes a very large barrier thickness (2.5 mm Pb) and converges to the actual value using a bisection method.

In practice, this takes approximately 50 iterations or less. Figure 3 shows the convergence of x as a function of iteration number. For the purpose of this paper, $\theta =0^{\circ }$, in accordance with NCRP Report 147 assumptions.

The same methodology is used to calculate unshielded air‐kerma rate and barrier thickness for the floor and ceiling. Instead of sampling across a line 0.3 m away from the barrier, RadShield samples a plane located at a user‐specified distance from the floor or ceiling. The unshielded air‐kerma rate for each exam is summed at each sampled point, and the barrier thickness is calculated iteratively as described above.

### B. Geometry‐based approach

In order to validate the results of RadShield, a second separate approach for finding the values and locations of maximum air‐kerma rates was developed. The geometry‐based method was designed to independently validate the locations of maximum air‐kerma rate found by RadShield. A series of equations was derived to yield the location of the point of maximum air‐kerma rate with respect to a reference location. Three general cases were considered: two point sources with two degrees of freedom, three point sources with two degrees of freedom, and three point sources with three degrees of freedom shielded by one barrier. The first two cases are analogous to shielding wall barriers and the last case is analogous to shielding floor and ceiling barriers.

**Figure 4:**
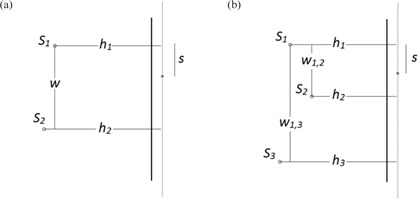
Geometry‐based approach with two degrees of freedom (a). Two isotropic sources with two degrees of freedom are shielded by a single barrier. A separate Java program samples the first derivative of the air‐kerma rate function across the sampled line to find its extrema. The air‐kerma rates at the extrema are found and the global maximum is reported. The process is repeated (b) for three sources with two degrees of freedom.

The methodology is described as follows. Suppose two point sources with strengths $S_{1}$ and $S_{2}$ are located at distances $h_{1}$ and $h_{2}$, respectively, from a line parallel to 0.3 m away (the sampled line) from a barrier (Fig. 4(a)). Let $S_{1}$ and $S_{2}$ be separated by a distance, w, in the dimension parallel to the sampled line. The air‐kerma rate at a distance, s, from the projection of $S_{1}$ onto the sampled line is given by the equation:

(6) $$K(s)=\frac{s_{1}}{{h_{1}}^{2}+s^{2}}+\frac{s_{2}}{{h_{2}}^{2}+{\left(w–s\right)}^{2}}$$

The first derivative of *K* with respect to *s* will yield the instantaneous rate of change of the air‐kerma rate as a function of s.

(7) $$\frac{dK}{ds}=-\frac{2S_{1}s}{{\left({h_{1}}^{2}+s^{2}\right)}^{2}}+\frac{2S_{2}(w-s)}{({h_{2}}^{2}+{{\left(w–s\right)}^{2})}^{2}}$$

Setting this equal to zero and solving for s yields local minima and maxima locations of the air‐kerma rate along the sampled line. Using a separate Java program, points are tested along the length of the sampled line at ūm increments for their closeness to zero. If $|\text{dK}/\text{ds}|<10^{-6}$, this is recorded as a root. The root and the kerma rate found at the location of the root are compared to the other calculated roots to determine the global maximum air‐kerma rate, and repeated.

Suppose another point source with strength $S_{3}$ is added and located at a distance $h_{3}$ from a line parallel to and 0.3 m away (the sampled line) from a barrier. Let $S_{1}$ and $S_{2}$ be separated by a distance $w_{1,2}$, and $S_{1}$ and $S_{3}$ be separated by a distance $w_{1,3}$ in the dimension parallel to the sampled line (Fig. 4(b)). The kerma rate along s is given by:

(8) $$K(s)=\frac{S_{1}}{{h_{1}}^{2}+s^{2}}+\frac{S_{2}}{{h_{2}}^{2}+{\left(w_{1,2}–s\right)}^{2}}+\frac{S_{3}}{{h_{3}}^{2}+{\left(w_{1,3}–s\right)}^{2}}$$

The first derivative of K yields the instantaneous rate of change of the air‐kerma rate as a function of s.

(9) $$\frac{dK}{ds}=-\frac{2S_{1}s}{{\left({h_{1}}^{2}+s^{2}\right)}^{2}}+\frac{2S_{2}(w_{1,2}-s)}{({h_{2}}^{2}+{{\left(w_{1,2}–s\right)}^{2})}^{2}}+\frac{2S_{3}(w_{1,3}-s)}{({h_{3}}^{2}+{{\left(w_{1,3}–s\right)}^{2})}^{2}}$$

The roots and their corresponding air‐kerma rates are again compared to those obtained using RadShield.

**Figure 5:**
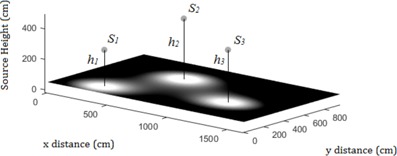
Geometry‐based approach with three degrees of freedom. A separate Java program is used to sample the 2D first derivative of the air‐kerma rate across the plane for its extrema. The extrema locations are entered into the air‐kerma rate function and the global maximum is found and the value reported.

For the case of three sources with three degrees of freedom, the air‐kerma rate function becomes a function of x and y (Fig. 5). Suppose three point sources with strengths $S_{1},S_{2}$, and $S_{3}$ are located at distances $h_{1},h_{2}$, and $h_{3}$, respectively, from a plane parallel to a barrier. Let $S_{1}$ and $S_{2}$ be separated by distances $x_{1,2}$ in x and $y_{1,2}$ in y, and let $S_{2}$ and $S_{3}$ be separated by distances $x_{1,3}$ in x and $y_{1,3}$ in y. The air‐kerma rate at a distance s_x_ from the projection of $S_{1}$ onto the x‐axis and s_y_ from the projection of $S_{1}$ onto the y‐axis is given by the equation

(10) $$K(s_{x},s_{y})=\frac{S_{1}}{{h_{1}}^{2}+{s_{x}}^{2}+{s_{y}}^{2}}+\frac{S_{2}}{{h_{2}}^{2}+{\left(x_{1,2}–s_{x}\right)}^{2}+{\left(y_{1,2}–s_{y}\right)}^{2}}+\frac{S_{3}}{{h_{3}}^{2}+{\left(x_{1,3}–s_{x}\right)}^{2}+{\left(y_{1,3}–s_{y}\right)}^{2}}$$

In this case, the first derivative with respect to s_x_ and s_y_ are

(11) $$\frac{\partial K}{\partial s_{x}}=\frac{2S_{1}s_{x}}{{\left({h_{2}}^{2}+{s_{x}}^{2}+{s_{y}}^{2}\right)}^{2}}+\frac{2S_{2}(x_{1,2}-s_{x})}{({h_{2}}^{2}+{\left(x_{1,2}–s_{x}\right)}^{2}+{{\left(y_{1,2}–s_{y}\right)}^{2})}^{2}}+\frac{2S_{3}(x_{1,3}-s_{y})}{({h_{3}}^{2}+{\left(x_{1,3}–s_{x}\right)}^{2}+{{\left(y_{1,3}–s_{x}\right)}^{2})}^{2}}$$

(12) $$\frac{\partial K}{\partial s_{y}}=\frac{2S_{1}s_{y}}{{\left({h_{2}}^{2}+{s_{x}}^{2}+{s_{y}}^{2}\right)}^{2}}+\frac{2S_{2}(y_{1,2}-s_{y})}{({h_{2}}^{2}+{\left(x_{1,2}–s_{x}\right)}^{2}+{{\left(y_{1,2}–s_{y}\right)}^{2})}^{2}}+\frac{2S_{3}(y_{1,3}-s_{y})}{({h_{3}}^{2}+{\left(x_{1,3}–s_{x}\right)}^{2}+{{\left(y_{1,3}–s_{x}\right)}^{2})}^{2}}$$

Points are tested by scanning the first derivative space at 0.1 mm increments for their closeness to zero. If $|\delta K/\delta s_{x}|<10^{-6}$ and $|\delta K/\delta s_{y}|<10^{-6}$, this is recorded as a root. The roots and the kerma rates found at the location of the roots are again compared to the value of maximum air‐kerma rate and its location obtained from RadShield.

In testing these three cases, source strengths ($S_{1},S_{2},S_{3}$), general orientation ($h_{1},h_{2},h_{3}$) and distances from each other (w, $x_{12},y_{12},x_{13},y_{13}$) are varied between 2 and 10 m. Each combination of constants is entered into both RadShield and the geometry‐based program and their percentage errors were found.

### C. Manual approach

For validation, an ABR certified diagnostic medical physicist performed shielding calculations for two clinical scenarios. The first scenario was a shielding design for two adjoining catheterization labs with a common control room. The second scenario was a shielding design for a radiographic and fluoroscopic room consisting of three exam types: chest radiographic exams, table top radiographic exams, and fluoroscopic exams. For each scenario, all the necessary information regarding X‐ray tube and patient locations, design goals and occupancy factors for all nearby rooms, workload distributions and floor plan scale were shared.

The physicist was asked to perform the calculations using the XRAYBARR program and was allowed to make simplifying assumptions within the scope of NCRP Report 147. The physicist only considered distances in one dimension, per the examples presented in NCRP Report 147. Careful attention was paid to make sure consistent geometry was maintained and that identical workload distributions were used. The physicist was asked to calculate the required barrier thicknesses for all materials covered in NCRP Report 147 and was not asked to perform the design with 0.079 cm lead thickness increments in mind, so as to provide for the most precise comparison between each of the compared methods. The maximum air‐kerma rates and barrier thicknesses for six materials were found by the physicist and compared to the same results found by RadShield. The percentage errors and absolute errors between results were found and compared.

## III. RESULTS

Semiautomated shielding design by RadShield was tested against two approaches: a geometry‐based approach and manual approach. Excellent agreement was observed between RadShield's approach and the geometry‐based approach. The maximum percentage error in calculated air‐kerma rate between the RadShield method and the geometry‐based method was 0.00017%, 0.00042%, and 0.00124% for two sources, three sources with two degrees of freedom, and three sources with three degrees of freedom, respectively (1, 2, 3). The largest separation distance in the point of maximum air‐kerma rate between the RadShield method and the geometry‐based method was 6.3 mm, 9.6 mm, and 14.0 mm for two sources, three sources with two degrees of freedom, and three sources with three degrees of freedom cases.

Good agreement was found between RadShield's approach and the manual approach in the adjacent catheterization labs scenario. The percentage error in calculated air‐kerma rate between RadShield and the manual approach ranged from 0.67% and 3.58% with an average of 1.60%. The absolute error in air‐kerma rate ranged from $0.031\;{\text{mGy wk}}^{-1}$ to $0.146\;{\text{mGy wk}}^{-1}$, with an average error of $0.069\;{\text{mGy wk}}^{-1}$. The percentage error in calculated lead barrier thickness ranged from 0.01% and 1.81%, with an average of 0.88%. The error in lead barrier thickness ranged from 0.010 mm to 0.156 mm, with an average error of 0.025 mm (Table 4).

**Table 1:** Comparison between RadShield approach and geometry‐based approach with two sources with two degrees of freedom.

*RadShield Approach*		*Geometry‐based Approach*		*Comparison*		
*Relative Location (m)*	*Air‐Kerma Rate* $\left(mGy\;wk^{-1}\right)$	*Relative Location (m)*	*Air‐Kerma Rate* $\left(mGy\;wk^{-1}\right)$	*Location Location % Error*	*Air‐Kerma Abs Error (m)*	*Rate % Error*
1.00E+00	8.64E−01	1.00E+00	8.64E−01	8.00E−06	8.00E−08	−1.42E−06
1.58E+00	7.07E−01	1.58E+00	7.07E−01	1.66E−01	2.62E−03	−4.76E−05
7.00E−01	1.09E+00	7.01E−01	1.09E+00	−1.47E−01	1.03E−03	−7.96E−06
1.22E+00	9.55E−01	1.22E+00	9.55E−01	3.24E−01	3.95E−03	−8.31E−05
1.50E+00	7.77E−01	1.50E+00	7.77E−01	3.56E−06	5.33E−08	−1.28E−06
2.52E+00	6.66E−01	2.52E+00	6.66E−01	3.98E−04	1.00E−05	−1.27E−06
7.40E−01	9.91E−01	7.46E−01	9.91E−01	−8.59E−01	6.36E−03	−1.76E−04
1.90E+00	8.81E−01	1.90E+00	8.81E−01	2.25E−01	4.27E−03	−5.95E−05

**Table 2:** Comparison between RadShield approach and geometry‐based approach with three sources with two degrees of freedom.

*RadShield Approach*		*Geometry‐based Approach*		*Comparison*		
*Relative Location (m)*	*Air‐Kerma Rate* $\left(mGy\;wk^{-1}\right)$	*Relative Location*	*Air‐Kerma Rate* $\left(mGy\;wk^{-1}\right)$	*Location % Error*	*Location Error (m)*	*Air‐Kerma Rate % Error*
2.00E+00	1.16E+00	2.00E+00	1.16E+00	4.50E−06	9.00E−08	−2.93E−06
2.56E+00	1.05E+00	2.57E+00	1.05E+00	−3.77E−01	9.64E−03	−4.27E−04
1.42E+00	1.34E+00	1.42E+00	1.34E+00	−3.29E−01	4.67E−03	−7.55E−05
1.82E+00	1.26E+00	1.81E+00	1.26E+00	3.07E−01	5.59E−03	−1.41E−04
3.00E+00	9.78E−01	3.00E+00	9.78E−01	2.00E−06	6.00E−08	−2.67E−06
3.64E+00	9.20E−01	3.64E+00	9.20E−01	8.52E−02	3.10E−03	−2.84E−05
1.52E+00	1.13E+00	1.51E+00	1.13E+00	5.07E−01	7.70E−03	−6.99E−05
2.72E+00	1.11E+00	2.73E+00	1.11E+00	−2.58E−01	7.02E−03	−1.65E−04
3.50E+00	7.71E−01	3.50E+00	7.71E−01	9.43E−02	3.30E−03	−3.74E−05
3.90E+00	7.23E−01	3.91E+00	7.23E−01	−1.56E−01	6.08E−03	−1.95E−04
6.20E−01	9.59E−01	6.13E−01	9.59E−01	1.20E+00	7.44E−03	−2.49E−04
3.40E+00	9.09E−01	3.40E+00	9.09E−01	−4.21E−02	1.43E−03	−8.30E−06
6.50E+00	7.71E−01	6.50E+00	7.71E−01	−5.08E−02	3.30E−03	−3.74E−05
6.62E+00	7.60E−01	6.61E+00	7.60E−01	1.38E−01	9.15E−03	−2.57E−04
2.80E−01	8.65E−01	2.83E−01	8.65E−01	−1.19E+00	3.34E−03	−8.02E−05
6.16E+00	8.72E−01	6.16E+00	8.72E−01	3.15E−02	1.94E−03	−1.72E−05

Agreement found between RadShield's approach and the manual approach in the R&F room was mixed. The percentage error in calculated air‐kerma rate between RadShield and the manual approach ranged from 4.40% and 110.467%, with an average of 28.48%. The absolute error in air‐kerma rate ranged from $0.036\;{\text{mGy wk}}^{-1}$ to $0.822\;{\text{mGy wk}}^{-1}$, with an average error of $0.247\;{\text{mGy wk}}^{-1}$. The percentage error in calculated lead barrier thickness ranged from 1.80% and 63.53%, with an average of 18.38%. The error in lead barrier thickness ranged from 0.015 mm to 0.160 mm, with an average error of 0.030 mm (Table 5).

**Figure 6:**
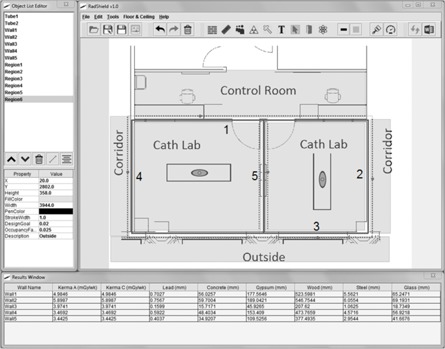
Completed RadShield design for the R&F room example. Nonadjacent rooms are sampled along lines 0.3 m inside their boundaries. Points at which the air‐kerma rate and barrier thickness were calculated are shown as small dark circles, while large dark circles show points of maximum calculated thickness for each wall. A star shows the point chosen to calculate shielding for the leftmost wall 4.

The R&F room was a more complicated scenario (Fig. 6). Three X‐ray sources with three different workload distributions were shielded for a busy floor plan. Nonadjacent rooms required consideration and calculations differed between the physicist and RadShield. Walls 1, 2, and 3 had good agreement with 5.82% error in air‐kerma rate and 3.46% error in lead barrier thickness. RadShield and the medical physicist selected the same approximate point to shield. The medical physicist shielded wall 4 to the design goal and occupancy of the corridor beyond the restroom, while RadShield shielded to the adjacent corridor in the lower left corner of the floor plan. This led to a small difference in required thickness for wall 4 (0.145 mm Pb vs. 0.195 mm Pb), resulting in a slightly under shielded barrier. Functionally, wall 4 shields part of the middle corridor because it is within the line of sight between the tabletop X‐ray sources and middle corridor. Due to distance differences between the corridor and the exam room, wall 4 barrier thickness should be calculated with respect to the corridor beneath the restrooms rather than the corridor on the other side of them. Although the manually calculated amount of lead thickness was insufficient to shield the exam room to the design goal, both RadShield and the physicist suggest rounding to the same prefabricated thickness, 0.079 cm.

**Table 3:** Comparison between RadShield approach and geometry‐based approach for three sources with three degrees of freedom.

*RadShield Approach*			*Geometry‐based Approach*			*Comparison*	
*X Location (m)*	*Y Location (m)*	*Air‐Kerma Rate* (${\text{mGy wk}}^{-1}$)	*X Location (m)*	*Y Location (m)*	*Air‐Kerma Rate* (${\text{mGy wk}}^{-1}$)	*Location Error (m)*	*Air‐Kerma Rate % Error*
2.64E+00	2.66E+00	9.77E−01	2.65E+00	2.65E+00	9.77E−01	1.41E−02	−6.27E−04
2.80E+00	2.82E+00	9.33E−01	2.81E+00	2.81E+00	9.33E−01	1.42E−02	−5.78E−04
2.32E+00	2.32E+00	1.08E+00	2.31E+00	2.31E+00	1.08E+00	1.23E−02	−3.12E−04
2.16E+00	2.80E+00	1.07E+00	2.16E+00	2.81E+00	1.07E+00	7.16E−03	−1.82E−04
3.56E+00	5.08E+00	7.62E−01	3.56E+00	5.08E+00	7.62E−01	3.26E−03	−2.54E−05
3.58E+00	5.16E+00	7.49E−01	3.57E+00	5.17E+00	7.49E−01	1.08E−02	−2.64E−04
2.40E−01	2.40E−01	8.93E−01	2.33E−01	2.33E−01	8.93E−01	1.02E−02	−6.73E−04
3.30E+00	5.12E+00	8.62E−01	3.30E+00	5.13E+00	8.62E−01	8.16E−03	−2.85E−04
4.10E+00	9.82E+00	5.82E−01	4.11E+00	9.82E+00	5.82E−01	5.95E−03	−2.80E−04
4.10E+00	9.84E+00	5.80E−01	4.11E+00	9.83E+00	5.80E−01	1.29E−02	−1.24E−03
6.00E−02	6.00E−02	7.86E−01	5.69E−02	6.43E−02	7.86E−01	5.30E−03	−2.47E−04
4.12E+00	9.74E+00	6.43E−01	4.12E+00	9.74E+00	6.43E−01	4.04E−03	−1.11E−04
5.80E+00	2.76E+00	7.75E−01	5.80E+00	2.76E+00	7.75E−01	4.60E−03	−8.57E−05
5.84E+00	2.82E	7.63E−01	5.85E+00	2.82E+00	7.63E−01	8.88E−03	−4.65E−04
2.60E−01	1.40E−01	8.72E−01	2.54E−01	1.30E−01	8.72E−01	1.17E−02	−9.95E−04
5.80E+00	4.96E+00	8.30E−01	5.80E+00	4.95E+00	8.30E−01	6.75E−03	−1.41E−04

**Table 4:** Air‐kerma rate and barrier thickness percentage errors between RadShield and manual approach: catheterization lab.

*Wall Number*	*Kerma Rate*	*Lead*	*Concrete*	*Gypsum*	*Wood*	*Steel*	*Glass*
1	0.67%	0.01%	2.83%	2.35%	1.18%	2.77%	1.25%
2	1.88%	0.50%	2.53%	2.03%	1.42%	2.35%	0.97%
3	3.58%	1.81%	0.52%	1.02%	4.52%	3.07%	0.19%
4	0.96%	1.27%	2.58%	2.10%	1.33%	2.62%	1.05%
5	0.90%	0.81%	2.33%	1.86%	1.59%	3.34%	1.08%
Average	1.60%	0.88%	2.16%	1.87%	2.01%	2.83%	0.91%

**Table 5:** Air‐kerma rate and barrier thickness percentage errors between RadShield and manual approach: R&F room.

*Wall Number*	*Kerma Rate*	*Lead*	*Concrete*	*Gypsum*	*Wood*	*Steel*	*Glass*
1	4.40%	5.77%	4.92%	4.74%	0.38%	8.13%	4.65%
2	7.58%	2.80%	0.21%	0.23%	2.88%	1.01%	0.97%
3	5.49%	1.80%	1.50%	1.41%	1.73%	15.50%	1.15%
4	24.06%	25.79%	21.92%	24.31%	14.55%	28.97%	23.26%
5	110.46%	63.53%	51.57%	57.16%	40.39%	72.45%	52.45%
6	18.91%	10.62%	7.55%	8.60%	8.45%	10.48%	8.46%
Average	28.48%	18.38%	14.61%	16.08%	11.40%	22.76%	15.16%

Large percentage errors in required lead thickness were observed for wall 5 (63.53% and 0.160 mm) and wall 6 (10.62% and 0.040 mm). The medical physicist overshielded these walls in both cases if results are compared with RadShield. The large percentage error is a result of the physicist only considering distance in one dimension, per the exemples provided by NCRP Report 147. RadShield automatically uses the triangulated distance based on the floor plan dimensions and user‐entered scale. There fore, RadShield's measurement reflects the more accurate expected distances from each source to the calculation point given the nonperpendicular geometry.

## IV. DISCUSSION

This work presents an improved methodology to radiation shielding design which performs many calculations beyond each barrier and reports the maximum calculated value of barrier thickness. RadShield's accuracy in finding the location of maximum air‐kerma rate was validated using a set of mathematical equations describing a finite number of isotropic X‐ray sources and scattering bodies. RadShield's accuracy in determining barrier thickness was tested against the thickness values found by an ABR‐certified medical physicist.

Air‐kerma rates and maximum air‐kerma rate locations obtained by RadShield agree with the geometry‐based equations to within 0.01% error and 1.4 cm. This is compelling empirical evidence that the geometry‐based equations and RadShield's methodology are correct and fundamentally valid. From the data, RadShield performs best when the air‐kerma rate is slowly varying because RadShield calculates across sampled lines beyond barriers. When the underlying distribution of sources and distances becomes more complicated, the point‐to‐point change in air‐kerma rate tends to increase and maximum point accuracy decreases. This variation increases when greater degrees of freedom are allowed such that falloff can occur in a second dimension. Nevertheless, the greatest deviation in the location of maximum air‐kerma rates between RadShield and the geometry‐based method is 14 mm and is certainly unlikely to be significant for clinical diagnostic radiology shielding calculations.

**Figure 7:**
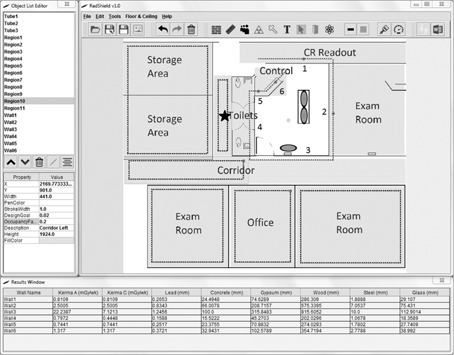
A completed RadShield shielding design and calculation for two adjacent catheterization labs. Small dots on the sampled line indicate points where air‐kerma rate and barrier thickness were calculated. Large dots on the sampled line indicate points of maximum required material thickness per wall. The object list editor to the left is used to enter shielding variables, such as design goal and occupancy factor, and to adjust colors and visual preferences. The results window below the workspace yields calculated unshielded air‐kerma rates (${\text{mGy wk}}^{-1}$) and barrier thickness (mm) for various materials.

Figure 7 shows the completed RadShield calculation for two adjacent catheterization labs. Each shaded room indicates a region with an occupancy adjusted design goal. RadShield loops through the regions for each calculation point and tests whether the calculation point is contained within the boundary of a region. When the correct region is found, RadShield extracts the design goal and occupancy factor information needed for the shielding calculation. Likewise, if a wall is within the line of sight of a calculation point and an X‐ray source, and the calculation point is 0.3 m from the wall, that wall's workload distribution is used for calculating unshielded air‐kerma rate and barrier thickness using its workload‐specific fitting parameters. For the wall that separates the two rooms, both the medical physicist and RadShield calculated required thickness from both sides of the wall and selected the maximum value. Maximum required thickness points for each wall are displayed as large, dark circles beyond each wall.

The adjacent catheterization labs example performed by RadShield and the medical physicist shows that RadShield handles relatively straightforward calculations without issue. The maximum thickness points occur in locations geometrically closest to the X‐ray sources, which is to be expected for uniform design goals and occupancy factors along the length of each wall. An important feature of RadShield is that it determines the orientation of X‐ray sources and barriers and generates lines 0.3 m beyond barriers, opposite from the X‐ray source. In this case, the middle wall is between two X‐ray sources and is simultaneously shielding two rooms. The air‐kerma rate contributions are not mixed; the calculation points on the left side of the middle wall only shield the X‐rays incident from the right and vice versa. Both RadShield and the medical physicist calculated the shielding requirements for both sides of the middle wall and shielded to and reported the maximum with good agreement.

The R&F room example shows that the medical physicist can miss the maximum thickness point. Figure 6 shows that the leftmost wall 4 should consider shielding for the main corridor rather than the corridor directly beyond the restrooms as the physicist had predicted (shown as a star). The large circle within the top right portion of the lower left corridor is the point of maximum calculated thickness.

Testing as many points as possible is most prudent no matter which computer program is used and was the basis for RadShield's design. RadShield's approach is a major improvement over current methods because its algorithm calculates at many points beyond each barrier and within nonadjacent rooms of interest. It has been shown that using a program like XRAYBARR and spreadsheets may not be sufficient for shielding design because the physicist has to know the most stringent point to shield *a priori* in order to guarantee all rooms meet their design goals, a difficult task for complicated geometries. RadShield might also be used to spot‐check manual calculations if needed.

The Java framework allows for other practical and convenient features beyond accuracy and thorough consideration of shielding design. The locations, colors, and properties of each drawn object may be saved in .xml format and loaded from disk. This gives the user the ability to change or modify shielding designs, either by entering new values or dragging objects to different locations, if the institution or hospital decides to repurpose a room in the floor plan or make adjustments to workload or exam geometry. Java library add‐ons allow the user to generate plots of air‐kerma rate across barriers and heat maps of air‐kerma rate on floor and ceiling barriers. Another library creates an automatically generated shielding report in a Microsoft Word document which summarizes the results and rounds lead thickness to the next highest 0.079 cm increment.

RadShield, as a stand‐alone program, brings greater possibility for current and future development. RadShield uniquely allows for easy inclusion of computerized tomography and positron related imaging incident on the same barriers because each X‐ray source and workload distribution is considered simultaneously in (4), (5). Oblique incidence is also integrated into RadShield as an option selectable by the user. A checkbox in a separate menu disables the NCRP 147 assumption that the angle of incidence, θ, is equal to 0°, and allows RadShield to calculate the angle between the barrier and the radiation line of sight. Therefore, the effective radiation path length can be considered and allow for the possibility of decreased shielding requirements and decreased cost.

Visual inspection of the 2D X‐ray exam distribution is not sufficient to accurately select the point of maximum air‐kerma rate or barrier thickness. RadShield can also eliminate the need for the physicist to select the most stringent point *a priori* by integrating a point‐by‐point calculation approach. This semiautomated approach reduces the likelihood of the medical physicist missing the maximum thickness point while also calculating thickness to a greater degree of accuracy than what would be found by following the examples contained in NCRP 147. It is understood that the medical physicist rounds to the nearest 0.079 cm increments of lead thickness; however, accurate calculation and consideration of all areas of interest is the appropriate starting point upon which to base more conservative shielding recommendations.

## V. CONCLUSIONS

RadShield was tested for its ability to determine the maximum air‐kerma rate point and the maximum shielding point. A comparison was made between two separate methods for independent validation. RadShield's point‐by‐point approach was shown to be effective in locating maximum points for a variety of exam distributions. A physicist will benefit from RadShield's approach to radiation shielding design because it reduces the possibility of missing the correct point to calculate required material thickness. This has been shown to be a problem when the physicist must consider regions of interest nonadjacent to the X‐ray room and when line of sight geometry becomes a factor. RadShield forms a core method as a stand‐alone program advancing the practical art of shielding, making it potentially more accurate and time‐efficient.

## COPYRIGHT

This work is licensed under a Creative Commons Attribution 3.0 Unported License.
